# Covalent and Site-Specific Immobilization of a Fluorogenic Sensor Protein on Cellulose-Based Paper for Detection of Lactate in Cell Culture Media

**DOI:** 10.3390/bios15100643

**Published:** 2025-09-28

**Authors:** Ingo Bork, Viktoria Höfling, Janine Becker, Markus Biesalski, Tobias Meckel, Harald Kolmar

**Affiliations:** 1 Institute for Organic Chemistry and Biochemistry, Technical University of Darmstadt, 64287 Darmstadt, Germany; 2 Macromolecular and Paper Chemistry, Chemistry Department, Technical University of Darmstadt, 64278 Darmstadt, Germany; markus.biesalski@tu-darmstadt.de (M.B.); tobias.meckel@tu-darmstadt.de (T.M.)

**Keywords:** lactate sensor, fluorogenic sensor, paper-based sensor, covalent paper modification, site-specific immobilization

## Abstract

Lactate is a key metabolite with applications ranging from monitoring training efficiency to early sepsis detection and monitoring the metabolic state of cell cultures. In this study, a paper-based lactate sensor utilizing a fluorescent readout was developed. Unlike common lactate dehydrogenase (LDH)-based methods, these sensors use a green fluorescent protein (GFP) or mApple-coupled lactate binding domain, which provides a fluorescent readout upon lactate binding. We demonstrate that immobilizing these proteins on paper does not affect their ability to bind lactate and produce a fluorescent readout, by monitoring lactate levels in the cell culture supernatant applying different cell culture conditions.

## 1. Introduction

Lactate is a pivotal metabolite that is produced through the glycolytic process under conditions of low oxygen [1]. The assessment of changes in the lactate levels of cell culture media offers insights into the status of the cells. Monitoring the lactate levels in cell culture applications is therefore of crucial importance in ensuring optimal productivity and growth [2]. Furthermore, lactate levels in different bodily fluids, including blood, sweat, and saliva, have been shown to provide diagnostic insights into various medical conditions and to assess the effectiveness of exercise regimens [3,4]. Lactate concentrations in the human bloodstream typically range from 0.5 to 1.5 mmol/L, while periods of stress or physical exertion have been shown to elevate lactate levels to 2.0–4.0 mmol/L [5]. In the context of septic shock, elevated lactate levels serve as a pivotal diagnostic indicator, given the established correlation between higher concentrations of lactate and the severity of the underlying disease [6]. A phenomenon, known as the Warburg effect, has been observed in the tumor microenvironment, where substantial increases in lactate levels are observed [7]. This highlights the potential of lactate monitoring as a vital tool in evaluating the therapeutic response of cancer patients. Current methodologies for lactate sensing can be categorized into two distinct approaches: enzyme-based methods that utilize lactate dehydrogenase (LDH) or lactate oxidase (Lox), and enzyme-free methods that depend on electrocatalytic materials, such as metal oxides and metal–organic frameworks (MOFs) [8,9,10,11]. The analytical outputs of these methods can be based on a variety of detection mechanisms, including colorimetric, fluorescence-based, and electrochemical readouts [12,13,14].

Due to its unique set of properties, paper has become a suitable material for the fabrication of sustainable and cost-effective diagnostic devices [15]. Its porous structure enables the transport of liquid samples through capillary forces alone, eliminating the requirement for external pumps [16]. Consequently, it is not surprising that numerous paper-based sensors have been developed for the detection of lactate in various fields of application. In the domain of healthcare and clinical monitoring, wearable paper-based patches have emerged as a novel tool for the assessment of lactate levels in sweat during physical exertion. Additionally, advancements in technology have led to the development of devices capable of rapidly detecting blood lactate levels in intensive care units. These devices offer potential benefits for healthcare professionals and patients alike [17,18]. Extracellular lactate levels are a critical component in the assessment of cell culture applications. The extracellular lactate concentration is an important biomarker that can indicate the metabolic state and overall healthiness of cancer cells used for disease models, as well as protein production [19,20]. The selection of an appropriate readout method is of crucial importance for these devices. Electrochemical methods offer a precise readout; however, they are more complicated and expensive to design and produce [21,22]. Conversely, colorimetric sensors are cost-effective to manufacture and the readout can be carried out using the naked eye or smartphone devices. However, the drawback is a lower level of sensitivity [23]. Devices that employ paper-based technology and utilize a fluorescent readout do not require the incorporation of electrodes while achieving highly sensitive quantitative readouts. Luongo et al. [24] developed methods to reduce background and optimize the fluorescent readout in paper-based devices. A notable disadvantage of this approach is the necessity of employing suitable light sources and detectors to obtain a readout [24].

To generate paper-based analytical devices for the detection of lactate, a reporter needs to be immobilized on the paper surface. Here, the most common reporter is LDH, but methods based on nanoparticles have been developed as well [25]. Next to these common LDH-based detection systems, Nasu et al. [26] have developed a set of red and green fluorescent lactate sensors. These sensors consist of a circular, permuted green fluorescent protein or circular permuted mApple which were inserted into a lactate binding protein. These constructs contain mutations leading to an inactive fluorophore that shows low fluorescence in the absence of lactate. Upon the binding of lactate, an allosteric structural rearrangement of the protein is triggered, resulting in the formation of an active fluorophore and a significant increase in fluorescence [26,27]. These sensors offer some advantages over the LDH-based assays in that they are insensitive towards the presence of LDH as well as pyruvate and other similar metabolites in the sample due to the selectivity of the employed lactate binding domain [26]. Additionally, LDH-based sensors require the presence of NAD^+^ and sometimes additional components to generate a colorimetric readout, while the sensors developed by Nasu et al. only require the presence of lactate, as well as calcium ions in the case of the green sensor [26,28].

A key challenge in the design of paper-based sensors is the immobilization of biomolecules on the paper surface. The often-employed method of adsorbing the biomolecule by printing of an aqueous solution suffers from reduced resolution caused by the capillary effect of paper as well as reduced activity caused by random orientation of the biomolecule and low stability due to the weak interactions with the paper surface. A preferred method is the immobilization of the biomolecule in a site-specific and covalent manner [29]. In previous work, we evaluated different strategies for this kind of covalent and site-specific immobilizations of biomolecules. We identified the SnoopTag/SnoopCatcher system developed by the Howarth and co-workers [30] as an efficient method for the immobilization of biomolecules onto cellulose-based papers that does not require chemical modification of the biomolecule itself [31]. This is achieved through the covalent binding of a modified version of the SnoopTag peptide to maleimide pre-modified paper, facilitated by a photocatalyzed Diels–Alder reaction. Subsequently, the desired biomolecule, equipped with the SnoopCatcher protein, is covalently attached to the paper-bound SnoopTag by a spontaneous formation of an isopeptide bond between an aspartate residue of the SnoopCatcher and a lysine residue of the SnoopTag [31]. In the present study, we have taken the sensors developed by Nasu et al. and genetically engineered them to be able to immobilize them to cellulose-based paper using our previously reported method based on the SnoopTag/SnoopCatcher system. To this end, a *C*-terminal fusion of the SnoopCatcher domain to the sensor protein was generated that was conjugated to commercially available filter paper pre-modified with the SnoopTag peptide. We were able to show that the immobilization of the sensors did not negatively impact their lactate binding capabilities. Furthermore, we showed that the generated paper-based lactate sensors can be used to assess the changes of extracellular lactate levels in two different cell culture applications. Here, the paper-based sensors were shown to be less susceptible to changes in the composition of the used cell culture medium, compared to a published LDH-based assay. Additionally, our sensors showed a higher sensitivity compared to the LDH-based assay.

## 2. Materials and Methods

Chemicals and solvents used for linker synthesis, analysis, and purification were purchased from BLDpharm (Shanghai, China), Iris Biotech (Marktredwitz, Germany), Carbolution (St. Ingbert, Germany), Jena Bioscience (Jena, Germany), Fisher Scientific (Waltham, MA, USA), Hycultec (Beutelsbach, Germany) and Carl Roth (Karlsruhe, Germany). Genes for plasmid generation were purchased from Twist Bioscience (South San Francisco, CA, USA) and PCR primers were purchased from Sigma-Aldrich (St. Louis, MO, USA). Whatman 1 paper was purchased from Sigma-Aldrich (St. Louis, MO, USA). Eucalyptus paper was made from bleached eucalyptus pulp. Gibco^TM^ RPMI 1640 medium and Gibco^TM^ Expi293^TM^ expression medium and Gibco^TM^ ExpiFectamine^TM^ 293 Transfection Kit were purchased from Fisher Scientific (Waltham, MA, USA).

### 2.1. Cell Culture

Hela cells were seeded into a 96-well plate in either RPMI medium + 1% Pen Strep or RPMI medium + 1% Pen Strep, 10% fetal bovine serum (FBS) (Sigma-Aldrich, St. Louis, MO, USA) at a cell density of 50,000 cells per well in 200 µL medium. After 24 h, 48 h, and 72 h, 150 µL of the supernatant was taken, centrifuged at 3000 rpm for 3 min, and frozen in liquid nitrogen. The frozen samples were stored at −20 °C until the assays were performed.

Expi293F cells were transfected with a plasmid for protein production using ExpiFectamine™ 293 reagent as transfection agent. The cells were cultivated in Expi293 expression medium, feeding the cells with ExpiFectamine™ 293 Enhancers 1 and 2 on day 1 after transfection. A total of 500 µL samples were taken after 24 h, 48 h, 72 h, 96 h, and 120 h. The samples were centrifuged at 3000 rpm for 3 min and frozen in liquid nitrogen. The frozen samples were stored at −20 °C until the assays were performed.

### 2.2. Confocal Laser Scanning Microscopy (CLSM)

Because it is not trivial to disintegrate the Whatman 1 filter paper into single paper fibers, CLSM studies were conducted using model fibers made from bleached eucalyptus pulp. The two interfaces are composed of cellulose surface chemistry, thus rendering the fiber modification method independent of the specific type of fiber utilized. Eucalyptus paper fibers modified with eLACCO2.1–SnoopCatcher were placed in a sample tray and MOPS buffer with 10 mM CaCl_2_ was added. Then, surplus buffer was removed and MOPS buffer containing 10 mM CaCl_2_ as well as 1 M sodium lactate was added and images were taken at different time points. Confocal microscopic analysis was performed using a Leica TSC SP8 confocal microscope (Leica Microsystems CMS GmbH, Wetzlar, Germany) equipped with a 20×/0.75 dry objective. GFP fluorescence was detected by excitation with a 488 nm laser and detected between 520 nm and 600 nm. Images were generated using Fiji, a distribution of ImageJ [32].

Time lapse sequences of the fluorescence intensity increase after lactate addition were recorded at a scan speed of 600 Hz with bidirectional scanning in the x-direction to achieve a temporal resolution of 433 ms per frame at a pixel count of 512 × 512. The image size is 1.55 × 1.55 µm. For the analysis, the “Plot Z-axis Profile” command in Fiji was used [32].

### 2.3. Methyl 4-((2-formyl-3-methylphenoxy)methyl)benzoic Acid (Photoenol)

The photoenol was available in our working group from previous research. Details on the synthesis are described in the literature [31].

### 2.4. SnoopTag-Containing Linker H-KLGDIEFIKVNK-AEEAc-AEEAc-K(photoenol)-G-NH_2_

The SnoopTag–Photoenol-linker was available in our working group from previous research. Details on the synthesis can be found in the literature [31].

### 2.5. Plasmid Generation of pET30-His6eLACCO2.1–SnoopCatcher–Twin-Strep-Tag and pET30-His6-R-iLACCO1.2–SnoopCatcher–Twin-Strep-Tag

For the production of pET30-His6eLACCO2.1–SnoopCatcher–Twin-Strep-Tag and pET30-His6-R-iLACCO1.2–SnoopCatcher–Twin-Strep-Tag, the genes were ordered at Twist Bioscience and cloned into a pET30 expression vector via Golden Gate assembly (GGA).

### 2.6. Protein Production and Purification

The expression plasmids were transformed into electrocompetent *Escherichia coli* (*E. coli*) T7 SOX cells by electroporation. The cells were then regenerated in Super Optimal Broth with catabolite repression (SOC) medium for 1 h at 37 °C, spread on dYT plates containing 0.1% (*v*/*v*) kanamycin, and incubated overnight at 37 °C. A single colony from the plate was then inoculated into 50 mL of dYT medium containing 0.1% (*v*/*v*) kanamycin and incubated overnight at 37 °C with gentle shaking. This preculture was then used to inoculate 1 L of Super Broth (SB) medium containing 0.1% (*v*/*v*) kanamycin to an OD600 of 0.1. The main culture was then subjected to incubation at 37 °C with gentle shaking until an OD600 of 0.5–0.8 was attained. At this point, protein production was induced by the addition of 1 mM isopropyl-β-D-1-thiogalactopyranoside (IPTG) and 5 g/l arabinose. Protein production was performed overnight at 25 °C with gentle shaking. The cells were then harvested by centrifugation, and the supernatant was discarded. The crude cell mass was resuspended in buffer (0.05 M Tris, 0.15 M NaCl, 0.001 M EDTA, pH 7.5). The cell suspension was sonicated to lyse the cells, and insoluble cell components were removed by centrifugation and sterile filtration. The protein of interest was isolated on a HisTrap HP column (1 mL, GE Healthcare, Chicago, IL, USA) using a linear gradient of imidazole. The constructs were further purified on a Strep-Tactin^®^XT 4Flow^®^ high capacity FPLC column (1 mL, IBA Lifesciences GmbH, Göttingen, Germany). The product fractions were subsequently pooled and dialyzed against phosphate-buffered saline (PBS) with a pH of 7.5 (molecular weight cutoff of 3.5 kDa). The protein sequences are presented in the Supporting Information, Sections S1.1–S1.2, and an SDS-Gel of the purified protein fractions is shown in Section 2.1.

### 2.7. Generation of SnoopTag-Modified Paper

The generation of maleimide functionalized paper and subsequent coupling of the SnoopTag–photoenol-linker was performed according to the protocol described in previous work [31]. Briefly, paper samples were first treated with 10% NaOH_(aq)_ to swell the paper fibers and remove loosely bound fines. Subsequently, Maleimidohexanoic acid was attached to the hydroxyl groups on the paper fibers by esterification with 1-Ethyl-3-(3-dimethylaminopropyl)carbodiimide (EDC) and 4-(Dimethylamino)pyridine (DMAP). The SnoopTag–photoenol-linker was then attached in a photocatalyzed Diels–Alder reaction between the maleimide groups on the paper fibers and the photoenol group on the linker.

### 2.8. Coupling of eLACCO2.1–SnoopCatcher and R-iLACCO1.2–SnoopCatcher to SnoopTag Pre-Modified Paper

The attachment of eLACCO2.1–SnoopCatcher and R-iLACCO1.2–SnoopCatcher to SnoopTag pre-modified paper was performed as a modified version of our previously published protocol [31]. Briefly, the paper samples were blocked with 3% (m/m) of bovine serum albumin (BSA) in PBS pH 7.5. Subsequently, the supernatant was removed and the samples were washed with Milli-Q grade water and PBS twice, respectively. A 4 µM solution of the respective protein was then added to the paper samples and the reaction mixture was incubated at 30 °C overnight under gentle agitation. The supernatant was removed and the samples were washed with 0.05% TWEEN 20 in 3-(*N*-Morpholino)propanesulfonic acid (MOPS) buffer (30 mM MOPS, 100 mM KCl, pH 7.2) and MOPS buffer twice. For the samples containing eLACCO2.1, the buffer was supplemented with 10 mM CaCl_2_ as this sensor is calcium dependent. The samples were stored in buffer at 4 °C until further use.

### 2.9. L-Lactate Dehydrogenase (L-LDH)-Based Assay in Solution

To evaluate the performance of our paper-based sensors, all samples were additionally subjected to an L-LDH-based lactate assay reported in the literature [28]. The assay was carried out as described by Schmiedeknecht et al. [28] Briefly, in a 96-well plate, 50 µL samples were mixed with 50 µL of the reaction buffer consisting of 17 µM nicotinamide adenine dinucleotide (NAD), 4.9 µM iodonitrotetrazolium chloride (INT), 0.25 µM 1-Methoxyphenazine methosulfate (M-PMS), and 6.9 nM L-LDH in 170 mM tris(hydroxymethyl)aminomethane (TRIS) buffer, pH 7.5. The reaction mixture was incubated for 1 h at ambient temperature in the dark, after which the reaction was quenched with 50 µL of 1 M acetic acid. Subsequently, the absorption at 490 nm was recorded on a CLARIOstar Plus plate reader (BMG LABTECH, Ortenberg, Germany). The cell culture supernatant samples were diluted 1/10 in MOPS buffer + 10 mM CaCl_2_ before performing the assay. For the calibration curve, RPMI medium, RPMI medium + 10% FBS, or Expi293^TM^ medium were diluted 1/10 in MOPS buffer + 10 mM CaCl_2_ and spiked with different amounts of sodium lactate.

### 2.10. Paper-Based Assay

A total of 50 µL of the sample was added to a Whatman 1 paper disk modified with eLACCO2.1 in a 96-well plate. The sample was then incubated for 10 min at room temperature with gentle agitation. Subsequently, the supernatant was removed, and the fluorescence of the paper samples was recorded using a CLARIOstar Plus plate reader (BMG LABTECH, Ortenberg, Germany) with an excitation wavelength of 480 nm and a 510 nm filter. The cell culture supernatant samples were diluted 1:10 in MOPS buffer containing 10 mM CaCl_2_ prior to the assay. To generate a calibration curve, RPMI medium, RPMI medium supplemented with 10% FBS, or Expi293™ medium were each diluted 1:10 in MOPS buffer supplemented with 10 mM CaCl_2_ and spiked with various concentrations of sodium lactate.

## 3. Results and Discussion

The lactate sensor proteins eLACCO2.1 and R-iLACCO1.2, which were generated by Nasu et al. [26] consist of the circular permuted fluorescent proteins GFP and mApple. In these sensors, a lactate binding domain was introduced into an intramolecular loop. This resulted in the generation of weakly fluorescent proteins, which exhibited a substantial increase in fluorescence upon lactate binding. Nasu et al. [26] successfully applied these sensors for the detection of changes in the intra- and extracellular lactate concentration of cells in vitro and in vivo. However, in their study, the researchers genetically modified the cells to either display the sensors on the cell membrane or produce the sensor intracellularly. We were curious to determine whether these sensors could be used for detecting lactate in general lactate-sensing applications when immobilized onto paper. Conventional lactate assays are frequently based on the conversion of lactate to pyruvate by the enzyme lactate dehydrogenase, with a simultaneous reduction in NAD+ to NADH. To achieve a fluorescent readout, this NADH production is coupled with a detection system, leading to a colorimetric or fluorescent readout. When utilized in an appropriate experimental setup, these assays demonstrate consistent efficacy in measuring lactate concentrations with a high degree of precision. However, this measuring principle is not without its drawbacks, including the interference caused by LDH or pyruvate in the sample. In the context of adapting this assay to a paper-based device, it is necessary to immobilize the LDH within the detection zone of the device. However, as previously mentioned, the readout is frequently accomplished by means of the generation of a colored small molecule. In a flow configuration, the molecule in question is not bound to the detection area, thereby complicating the readout process. A further issue is that these assays necessitate the presence of multiple components, including LDH, NAD+, and the compounds required to generate the desired readout. The samples are thus required to demonstrate compatibility with the addition of these compounds.

The present study hypothesized that the generation of paper-based tests with fluorogenic sensor proteins would offer certain advantages over the LDH-based approach. Specifically, these sensors do not necessitate the incorporation of supplementary components, with the exception of Ca^2+^ in the context of eLACCO2.1. The fluorescent readout can be confined to the detection zone, as the protein itself functions as the fluorophore.

**Figure 1:**
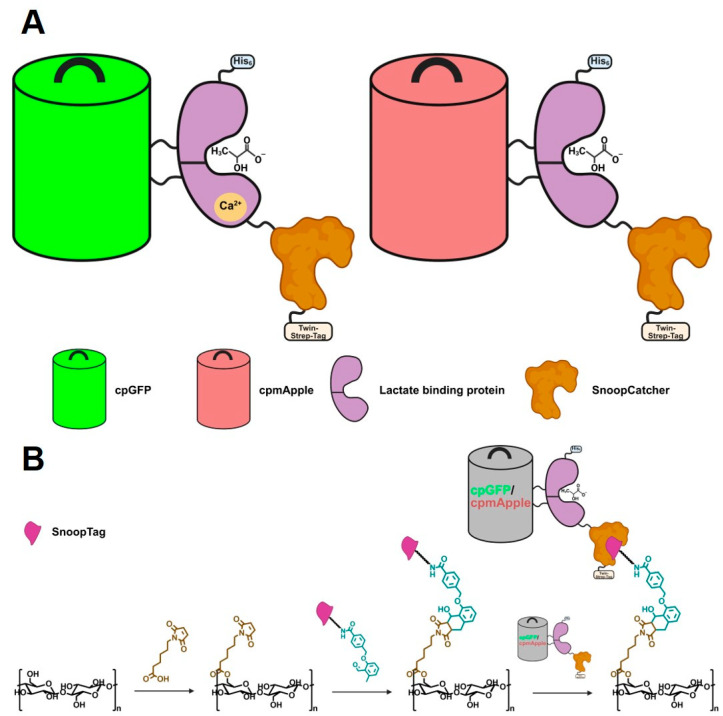
(**A**) The proteins eLACCO2.1 (left) and R-iLACCO1.2 (right) are shown with the N-terminally attached hexahistidine-tag as well as the C-terminally attached SnoopCatcher and Twin-Strep-Tag. (**B**) Coupling scheme for the sequential attachment of maleimidohexanoic acid, photoenol–SnoopTag-linker, and the lactate sensor onto cellulose-based paper.

The immobilization of the sensors was accomplished through the implementation of our previously developed method for the site-specific covalent attachment of proteins to cellulose-based paper [31]. The SnoopTag/SnoopCatcher-mediated approach was selected for this purpose, whereby the SnoopTag peptide is first attached to the paper fibers, and then the protein of interest is immobilized via its fusion with the SnoopCatcher sequence. To this end, the SnoopCatcher sequence was genetically attached to the *C*-terminus of the eLACCO2.1 and R-iLACCO1.2. Furthermore, a *N*-terminal hexahistidine-tag and a *C*-terminal Twin-Strep-Tag were incorporated to facilitate the purification of the proteins post-production in *E. coli*. Figure 1 presents a schematic representation of the constructs that were generated, as well as the immobilization process on paper.

**Figure 2:**
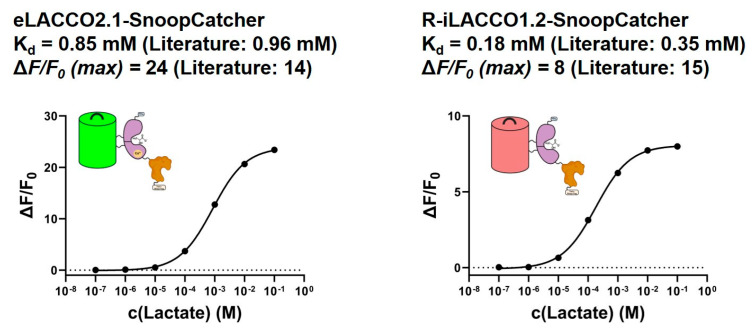
The lactate binding curves for SnoopCatcher-modified eLACCO2.1 (**left**) and R-iLACCO1.2 (**right**) are shown. Error bars are the result of two individual measurements. Literature reference values from from Nasu et al. [26].

After confirming the capacity of the SnoopCatcher-modified variants of eLACCO2.1 and R-iLACCO1.2 to be produced as soluble protein, an evaluation was conducted to determine whether the modified sensors retained their capacity to bind lactate and demonstrate a fluorescent response. To this end, the sensor protein absorption and emission maxima were initially examined in the presence and absence of 100 mM lactate. The findings of this study demonstrated comparable values to those reported in the relevant literature [26] (see Supporting Information Section S2.2). Subsequently, an evaluation was conducted to measure the maximum fluorescence response, denoted as the respective ΔF/F_0_ values, as well as the Kd with respect to lactate binding of the sensors in solution (see Figure 2).

The findings demonstrate that the *C*-terminal attachment of the SnoopCatcher moiety did not exert a substantial influence on the Kd of the sensors. The maximum fluorescence in relation to the baseline fluorescence, indicated by the ΔF/F0 values, exhibited an increase for the green sensor eLACCO2.1–SnoopCatcher. Conversely, R-iLACCO1.2–SnoopCatcher demonstrated a decline in this metric.

**Figure 3:**
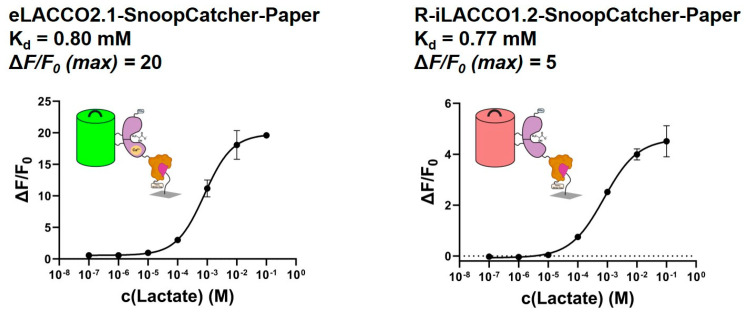
The lactate binding curves for the paper-bound sensors eLACCO2.1–SnoopCatcher (**left**) and R-iLACCO1.2–SnoopCatcher (**right**) are shown. Error bars are the result of two individual measurements.

Consequently, each sensor was conjugated to SnoopTag-pre-modified paper, as previously described, and the lactate binding capabilities of the resulting paper-based sensors were assessed (Figure 3).

The paper-based sensors demonstrate properties consistent with those of proteins in solution, exhibiting comparable Kd values and slightly diminished ΔF/F0 values. Consequently, it can be concluded that the adaptation of the fluorescent lactate binders eLACCO2.1 and R-iLACCO1.2 was successful. Due to the higher ΔF/F0 values obtained for the green sensor, eLACCO2.1–SnoopCatcher was selected for further experimentation.

**Figure 4:**
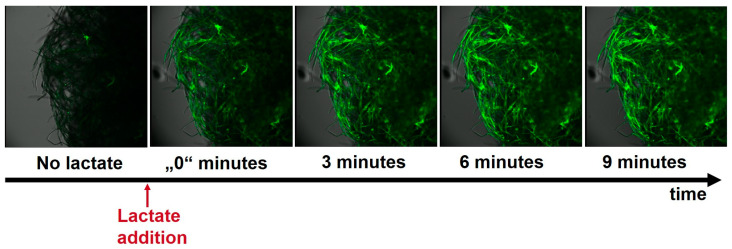
Eucalyptus paper fibers were modified with the sensor eLACCO2.1–SnoopCatcher. The fibers were then treated with 100 mM lactate, and the fluorescent response was analyzed at different time points using confocal microscopy. The term “0” minutes is used to denote the initial measurement taken immediately following the addition of lactate. The measurement was conducted approximately 30 s after the addition of lactate.

To evaluate whether the observed change in fluorescence could be tracked using confocal microscopy, we modified short eucalyptus paper fibers with the green sensor. We then observed the paper fibers at different time points after lactate addition to determine the speed of the fluorescent response. The results are shown in Figure 4.

A rapid response to the introduction of lactate was observed, indicated by a distinct increase in fluorescence immediately following the addition of lactate. Beyond the three-minute mark, a negligible rise in fluorescence was detected. To further investigate the kinetics of the lactate response, the increase in fluorescence in the first 400 s following lactate addition was measured by taking a video at a frame rate of 2.3 frames per second (see Supporting Information file 2). This finding indicated an almost immediate response following the addition of lactate. The mean fluorescence of each frame was calculated, yielding a half-maximal fluorescence intensity at 61.3 s (±6.1 s) and a maximum fluorescence intensity reached at approximately 4–5 min (see Supporting Information Section S4). In order to obtain preliminary data regarding the long-term stability of the paper-bound sensor, the sensor-modified fibers were stored at 4 °C in the dark in a buffer solution. After six months, the fibers were retested under similar conditions (data shown in the Supporting Information Section S4). These findings indicate that after a period of six months during which the fibers were stored, the addition of lactate to the solution was able to elicit a robust fluorescence response from the fibers. Subsequent to the storage period, a maximum ΔF/F0 value of 6.75 was obtained, in comparison to a maximum of 5.24 prior to storage, demonstrating a slight increase following storage. Consequently, it can be assumed that no degradation or inactivation of the sensor has occurred, even after extended storage periods. The observed increase in the maximum ΔF/F0 value is attributable to the elevated F0 value detected in the initial experiments. One potential explanation for this phenomenon is that a small percentage of the sensors may have been present in an active lactate-bound state due to residual lactate from the production in *E. coli* under semi-anaerobic conditions. Following extended storage in a buffer solution, it is likely that this lactate has undergone dissociation, thereby leading to a reduction in its F0 value. This finding aligns with the preliminary investigations conducted on the reusability of the sensors, wherein it was observed that after subjecting the soluble sensors to dialysis in a lactate-bound state for a period of three days, a decline in fluorescence intensity was detected. However, this decline did not attain the levels observed prior to the addition of lactate. These observations are consistent with the high off rate of 20 s reported in the existing literature [26].

**Figure 5:**
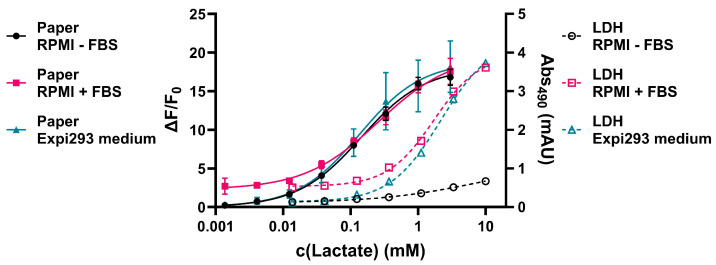
Standard curves for the determination of lactate concentrations in different cell culture media. The data for the paper-based assay is shown as ΔF/F_0_ values on the left y-axis, whereas the data for the LDH-based assay [28] is shown as absorbance at 490 nm on the right y-axis. The standard curves were fitted to a four-parameter logistic curve. Error bars are the result of three individual measurements (two for “Paper Expi293F^TM^”). Cell culture media were diluted 1:100 in the paper-based assay and 1:10 in the LDH-based assay.

To test whether our sensor could be used in a more complex environment, we decided to test it towards the tracking of lactate levels in two different cell culture applications. Prior to the cell assays, we examined the cell culture media spiked with different concentrations of sodium lactate to generate standard curves for the determination of the lactate concentration as well as to assess the influence of the contents of the cell culture media on the LDH-based assay in comparison to the paper-based fluorescence assay (Figure 5).

Notably, RPMI medium containing FBS showed an increased fluorescence readout at very low lactate supplementation. This can be explained by the lactate present in FBS [33]. At higher concentrations, the RPMI medium containing FBS yields similar fluorescence readouts to the RPMI medium without FBS as well as the Expi293 expression medium in the paper-based assay. The F_0_ values used for the calculation of the ΔF/F_0_ values represent the background fluorescence of the paper-based sensors in MOPS buffer. The fact that the bottom values are close to 0 for the RPMI medium and Expi293 medium indicate that the components of these media do not cause any intrinsic background fluorescence. Together with the comparable top values for the paper sensors in all of the media, this suggests that except lactate, the components of the media do not influence the fluorescent readout of the paper-based sensors. In contrast, in the LDH-based assay, the RPMI medium supplemented with FBS as well as the Expi293 medium show a much higher top value compared to the RPMI medium without FBS. This suggests that the LDH-based assay is affected by other components of the FBS and the Expi293 medium. These could potentially be sodium pyruvate or LDH itself being part of the medium [34]. Additionally, the EC_50_ values of the paper-based sensor are circa 10-fold lower compared to the LDH-based assay, showcasing the increased sensitivity of the paper-based sensor. Based on this data, the lower limit of detection of the paper-based sensor can be estimated to be in the 0.001–0.01 mM concentration range, as indicated by the lower plateau of the standard curves.

**Figure 6:**
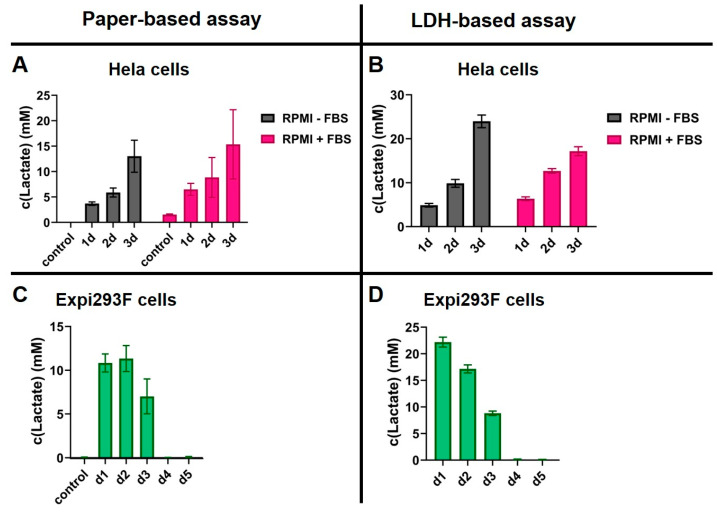
Lactate concentration in the supernatant of Hela and Expi293F cells at different time points as determined by either the paper-based assay (**A**,**B**) or the solution-based assay (**C**,**D**). Error bars are the result of three individual measurements. Controls refer to sensors treated only with the respective medium. Cell culture supernatants were diluted 100-fold in the paper-based assay and 10-fold in the solution-based assay. MOPS buffer with 10 mM Ca^2+^ was used to dilute the samples.

These standard curves were subsequently used to determine changes in the lactate levels of two different cell types. Specifically, adherent Hela cells were seeded in RPMI medium with or without FBS and cultivated for 3 days. A sample was taken from the supernatant after 1, 2, or 3 days and the lactate concentration was determined using our paper-based sensor, or an LDH-based assay [28]. Similarly, Expi293F cells were transfected with DNA for antibody production and the lactate levels were tracked during the production for 5 days in Expi293 expression medium. Again, a sample was taken each day, and the lactate level was determined using the paper-based and the solution-based methods. The results of these assays are shown in Figure 6.

For the adherent Hela cells, both assays detected an increase in lactate levels from day 1 to day 3. The calculated concentrations were in a similar range for both assays, with the paper-based assay yielding slightly lower concentrations overall. The standard deviations for the paper-based assay are notably higher. This phenomenon may be attributed to inadequate and heterogeneous loading of the sensor on the paper discs. It is recommended that subsequent optimization of the immobilization process be undertaken to reduce these errors. A notable benefit of the paper-based sensor is its independence from the components present in the cell culture media, with the exception of lactate, as previously discussed. This experimental design enabled the quantification of the lactate concentration in the RPMI medium with 10% FBS by utilizing the standard curve of RPMI medium devoid of FBS, as illustrated in the control column of part A in Figure 5. In the LDH-based assay, the standard curve is heavily influenced by the addition of FBS. Consequently, the standard curve of RPMI medium without FBS is not applicable for the calculation of the concentration in the RPMI medium containing 10% FBS. Furthermore, given the assay’s sensitivity to the addition of FBS, the validity of determining concentrations in the RPMI medium containing FBS remains uncertain.

During the process of antibody production in Expi293F cells, both assays demonstrate a surge in lactate concentration on days 1 and 2, subsequently followed by a decline in lactate levels until day 3, when complete lactate depletion is attained. This shift in lactate concentration is consistent with the existing literature, which reports a transition from aerobic metabolism to anaerobic metabolism following an initial peak in lactate concentrations [20]. During anaerobic metabolism, the lactate is used as an energy source subsequent to the depletion of the initial glucose reserves. In this experiment, the paper-based assay revealed a peak in lactate concentrations on day 2, while the LDH-based assay exhibited this peak on day 1. Given that a feeding step was conducted on day 1, a peak on day 2 would be expected; however, further investigation is necessary to determine the reason for the observed difference in the two assays. Furthermore, the LDH-based assay has been demonstrated to detect significantly higher lactate concentrations in the supernatant of Expi293F cells in comparison to the paper-based assay. As previously discussed, the Expi293 medium has a significant impact on the readout of the LDH-based assay, similar to the effect observed with FBS. Therefore, with regard to this assay, there is still doubt about the validity of the standard curve for determining lactate concentrations in Expi293 medium.

Despite the higher standard deviations observed in the collected data of the paper-based assay, our findings suggest that, particularly in the context of comparing the results of experiments conducted in different cell culture media, the paper-based assay can more accurately depict variations in lactate levels. This hypothesis is based on the findings that the paper-based assay is not influenced by components of the cell culture media other than lactate itself. Therefore, this approach enables the utilization of a universal calibration curve, derived from a lactate-spiked buffer solution, for the assessment of lactate concentrations across diverse cell culture media.

## 4. Conclusions

In the present study, we successfully genetically attached the SnoopCatcher moiety, along with suitable purification tags, to the fluorescent lactate sensors eLACCO2.1 and R-iLACCO1.2. The resulting constructs, His6-eLACCO2.1–SnoopCatcher–TwinStrep and His6-R-iLACCO1.2–SnoopCatcher–TwinStrep, exhibited comparable affinity for lactate binding and similar fluorescence increases compared to the data reported in the literature for the parental constructs. The generated constructs were successfully immobilized on Whatman 1 filter paper that had been pre-modified with the corresponding SnoopTag peptide. The resulting paper-based sensors retained their affinity for lactate binding, while simultaneously demonstrating only a slight decrease in the observed ΔF/F0 values. The change in the fluorescence of the green sensor could easily be detected using confocal microscopy, revealing an almost immediate increase in fluorescence after the addition of lactate. The fluorescent response of the paper-based sensor was found to be unaffected by the components of common cell culture media. These components had previously been identified as causing problems in a common LDH-based assay [34]. The high sensitivity of the generated paper-based sensors allows for the measurement of highly diluted samples, further reducing the influence of the initial media and minimizing the amount of sample required. These paper-based sensors were employed to monitor changes in lactate levels in the extracellular fluid of two prevalent cell lines, thereby demonstrating the sensors’ reliability and adaptability. Presently, the developed sensor demonstrates an elevated standard deviation in the context of cell culture experiments. This discrepancy can be attributed to variations in sensor loading resulting from the manual fabrication process and the limited sample size employed in this study. It is anticipated that the observed heterogeneity in the fluorescent readout will undergo a substantial improvement through the implementation of a more sophisticated and automated production process. It is hypothesized that these fluorescent sensors could offer certain advantages in applications where the detection of lactate in complex sample preparations is necessary and devices with immobilized sensors are desired.

## Data Availability

The original contributions presented in this study are included in the article/Supplementary Materials. Further inquiries can be directed to the corresponding author.

## Supplementary Materials

The following supporting information can be downloaded at: https://www.mdpi.com/article/10.3390/bios15100643/s1, Table S1: Additional data. Analysis of protein production, protein sequences as well as absorption and emission spectra of the generated proteins. Figure S1: SDS-Gel of the purified constructs. For both constructs a band at the expected molecular weight was observed. The Color Prestained Protein Standard, Broad Range (10–250 kDa) protein marker from New England Biolabs was used as protein marker; Figure S2: A, B: Absorption spectra of eLACCO2.1-SnoopCatcher and R-iLACCO1.2-SnoopCatcher, respectively. C, D: Emission scans of eLACCO2.1-SnoopCatcher and R-iLACCO1.2-SnoopCatcher, respectively; Figure S3: The raw fluorescence and absorbance data for the paper-based (A, C) and LDH-based (B, D) assay respectively, are shown. Error bars are the result of three individual measurements. Controls refer to sensors treated only with the respective medium. Cell culture supernatants were diluted 100-fold in the paper-based assay and 10-fold in the solution-based assay. MOPS buffer with 10 mM Ca2+ was used to dilute the samples; Figure S4: Confocal microscopy images taken of sensor-modified eucalyptus fiber at different timepoints after lactate addition and the maximum ΔF/F0–values determined by analyzing the mean fluorescence of the images in Fiji image J. A: Initial data obtained within a few days of the modification of the fibers. B: Data obtained after prolonged storage of the fibers for 6 month; Figure S5: The increase in fluorescence after lactate addition during the first 400 seconds is shown. 3 series were measured on different paper fibers to calculate the time at which the half-maximal fluorescence intensity is reached; Video S1: Kinetics of fluorescence increase after lactate addition.

## Abbreviations

The following abbreviations are used in this manuscript:

BSA	Bovine serum albumin
CLSM	Confocal laser scanning microscopy
E. coli	Escherichia coli
DMAP	4-(Dimethylamino)pyridine
EDC	1-Ethyl-3-(3-dimethylaminopropyl)carbodiimide
FBS	Fetal bovine serum
LDH	Lactate dehydrogenase
Lox	Lactate oxidase
GFP	Green fluorescent protein
GGA	Golden Gate assembly
INT	Iodonitrotetrazolium chloride
IPTG	Isopropyl-β-D-1-thiogalactopyranoside
MOFs	Metal–organic frameworks
M-PMS	1-Methoxyphenazine methosulfate
MOPS	3-(N-Morpholino)propanesulfonic acid
NAD	Nicotinamide adenine dinucleotide
PBS	Phosphate-buffered saline
SB	Super Broth
SOC	Super Optimal Broth with catabolite repression
TRIS	Tris(hydroxymethyl)aminomethane
