# Supplementary material for: Covalent and Site-Specific Immobilization of a Fluorogenic Sensor Protein on Cellulose-Based Paper for Detection of Lactate in Cell Culture Media
Source: Biosensors (Basel). 2025 Sep 28;15(10):643. doi: 10.3390/bios15100643 (PMC12562644; doi:10.3390/bios15100643)
Supplement: Supplementary file 1 [file biosensors-15-00643-s001.zip › Analysis of protein production, protein sequences as well as absorption and emission spectra of the generated proteins. (PDF).pdf]

Electronic Supplementary Information

Covalent and site-specific immobilization of a fluorogenic sensor protein on cellulose-based paper for detection of lactate in cell culture media

Ingo Bork [1], Viktoria Höfling [1], Janine Becker [1], Markus Biesalski [2], Tobias Meckel [2], and Harald Kolmar [1,]*

[1] Institute for Organic Chemistry and Biochemistry, Technical University of Darmstadt, 64287 Darmstadt, Germany

[2] Macromolecular and Paper Chemistry, Chemistry Department, Technical University of Darmstadt, 64278, Darmstadt, Germany

** Correspondence: Harald.Kolmar@TU-Darmstadt.de*

Table of contents

1 Protein sequences 3
1.1 His6-eLACCO2.1-SnoopCatcher-TwinStrep 3
1.2 His6-R-iLACCO1.2-SnoopCatcher-TwinStrep 4
2 Protein analytical data 5
2.1 SDS-Gel of the modified sensors 5
2.2 Absorption spectra and emission scans 6
3 Raw data for the paper-based and solution-based lactate assays 7
4 Analysis of the fiber-bound sensor after prolonged storage 8

1 Protein sequences

1.1 His6-eLACCO2.1-SnoopCatcher-TwinStrep

Protein-Sequence:

```
        10         20         30         40         50         60
MMAHHHHHHG SMFSPLAVAQ ARRYRWRIQT AWDAGTVGYS LFQKFTERVK ELTDGQLEVQ
        70         80         90        100        110        120
PFPASAVVGT FDMFDAVKTG VLDGMNPFTI YWAGRMPVTA FLSSYALGLD RPDQWETWFY
       130        140        150        160        170        180
SLGGLDNARR AFAEQGLFYV GPVQHDLNTI HSKKPIRRFE DFKGVKLRVP GGMIAEVFAA
       190        200        210        220        230        240
AGASTVLLPG GEVNPALERG VIDWSHNVHI MADKQRNGIK ANFEIRHSTE DGGVQLAYHY
       250        260        270        280        290        300
QQNTPIGDGP VLLPDNHYLS TQTKLTKDPN EKRDHMVLLE YVTAAGITRG MDELYKGGTG
       310        320        330        340        350        360
GRMVSKGEEL FTGVVPILVE LDGDVNGHKF SVSGEGEGDA TSGKLSLKFI STTGKLPVPW
       370        380        390        400        410        420
PTLVTTLTYG VQCFSRYPDH MKQHDFFKSA MPEGYIQERT IFFKDDGNYK TRAEVKFEGD
       430        440        450        460        470        480
TLVNRIELKG IDFKEDGNIL GHKLEYSFND GGAADFVGPA VNYNLGFHQV AKYIIMGPPE
       490        500        510        520        530        540
TPAIHQPVDL MDFTINLNRW RSLPKPLQER FIAAVHEYSW IHYAGIQKAN LEAWPKYRQA
       550        560        570        580        590        600
GVEVIRLSNE DVRKFRRLAI PIWFKWAKMD NYSREAFASQ LEYMKGIGYV TDEELKGLGL
       610        620        630        640        650        660
GGGGSGGGGS GGGGSKPLRG AVFSLQKQHP DYPDIYGAID QNGTYQNVRT GEDGKLTFKN
       670        680        690        700        710        720
LSDGKYRLFE NSEPAGYKPV QNKPIVAFQI VNGEVRDVTS IVPQDIPATY EFTNGKHYIT
       730        740        750        760
NEPIPPKGSA WSHPQFEKGG GSGGGSGGSA WSHPQFEK
```

Cyan: His$_6$-tag   Green: eLACCO2.1-sequence

Magenta: SnoopCatcher-sequence   Orange: Twin-Strep-tag

1.2 His6-R-iLACCO1.2-SnoopCatcher-TwinStrep

Protein-Sequence:

```
        10         20         30         40         50         60
MMAHHHHHHG SMEQNIVQPL KTLMGDDPDY SFDILEARYA IEASTAWYAA LRATPGDKEK
        70         80         90        100        110        120
IQLCFEATRS EDPDIASQAD VRFHLAIAEA SHNIVLLQTM RGFFDVLQSS VKHSRQRMYL
       130        140        150        160        170        180
DWVSERMFPE DGALKSEIKK GLRLKDGGHY AAEVKTTYKA KKPVQLPGAY VVDIKLDIVS
       190        200        210        220        230        240
HNEDYTIVEQ CERAEGRHST GGVDELYKGG TGGSLVSKGE EDDMAIVKEF MRFKVHMEGS
       250        260        270        280        290        300
VNGHEFEIEG EGEGRPYEAF QTAKLKVTKG GPLPFAWDIL SPQFMYGSKA YIKHPADIPD
       310        320        330        340        350        360
YFKLSFPEGF RWERVMYFED GGIIHVNQDS SLQDGVFIYK VKLRGTNFPP DGPVMQKKTM
       370        380        390        400        410        420
GWEARPPVYG QLTEQHQAVI DAIFAGDADG ARKAMMAHLS FIHTTMKRFD GDQARHARIS
       430        440        450        460        470        480
RLPGEHNEHS REKKAGGGGS GGGGSGGGGS KPLRGAVFSL QKQHPDYPDI YGAIDQNGTY
       490        500        510        520        530        540
QNVRTGEDGK LTFKNLSDGK YRLFENSEPA GYKPVQNKPI VAFQIVNGEV RDVTSIVPQD
       550        560        570        580        590
IPATYEFTNG KHYITNEPIP PKGSAWSHPQ FEKGGGSGGG SGGSAWSHPQ FEK
```

Cyan: His_6-tag Red: R-iLACCO1.2-sequence

Magenta: SnoopCatcher-sequence Orange: Twin-Strep-tag

2 Protein analytical data

2.1 SDS-Gel of the modified sensors

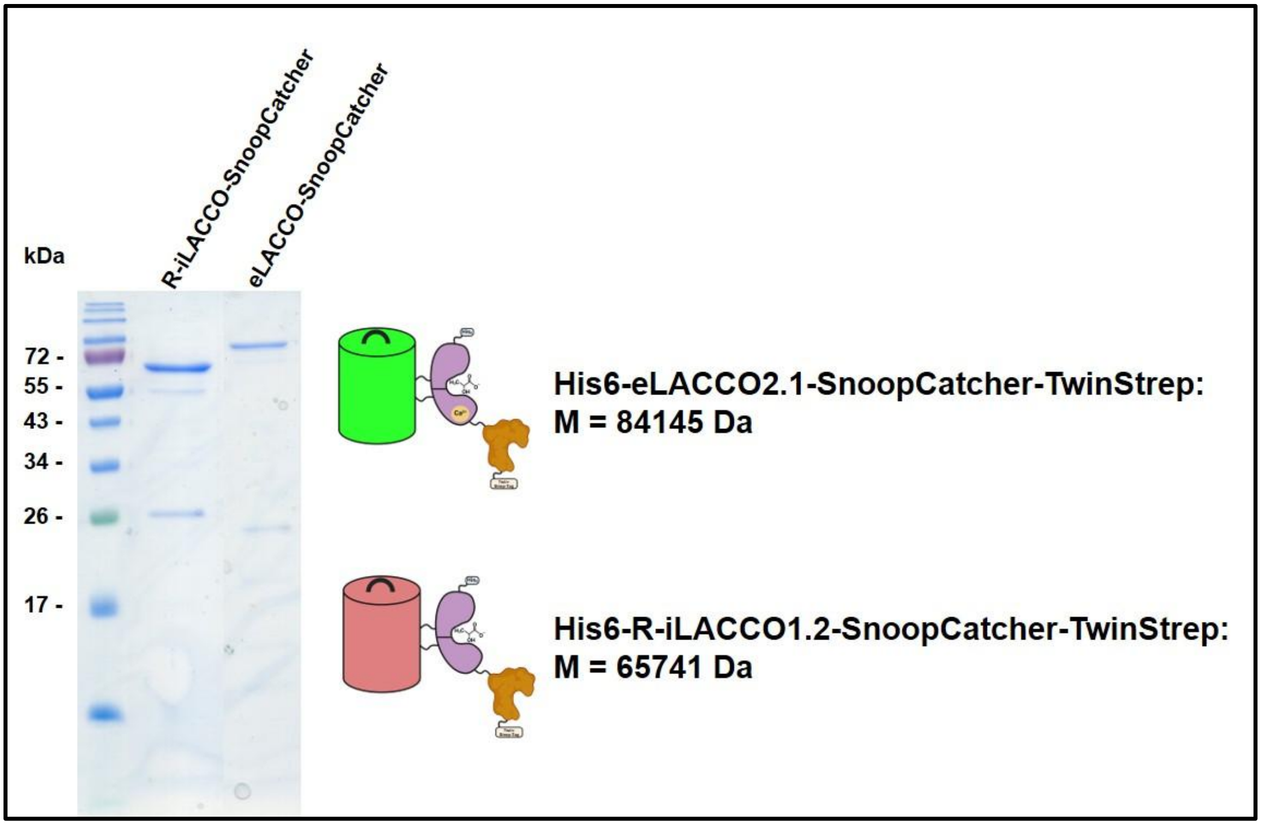

Figure S 1: SDS-Gel of the purified constructs. For both constructs a band at the expected molecular weight was observed. The Color Prestained Protein Standard, Broad Range (10–250 kDa) protein marker from *New England Biolabs* was used as protein marker.

2.2 Absorption spectra and emission scans

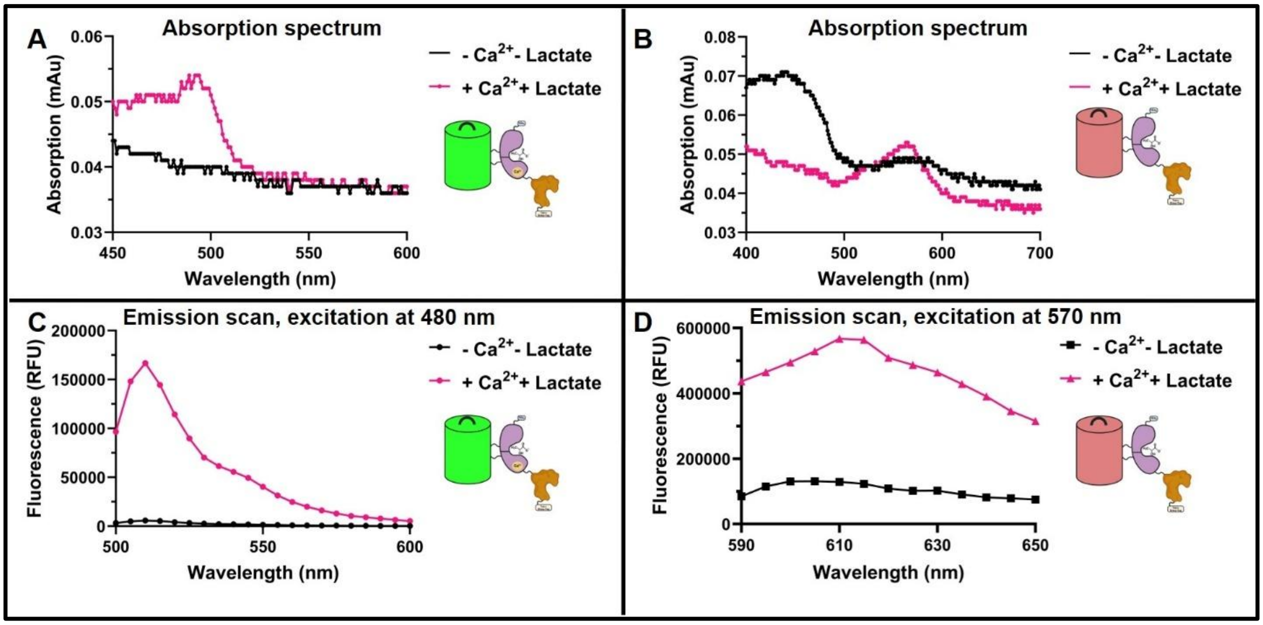

Figure S 2: ***A, B****: Absorption spectra of eLACCO2.1-SnoopCatcher and R-iLACCO1.2-SnoopCatcher, respectively.* ***C, D****: Emission scans of eLACCO2.1-SnoopCatcher and R-iLACCO1.2-SnoopCatcher, respectively.*

3 Raw data for the paper-based and solution-based lactate assays

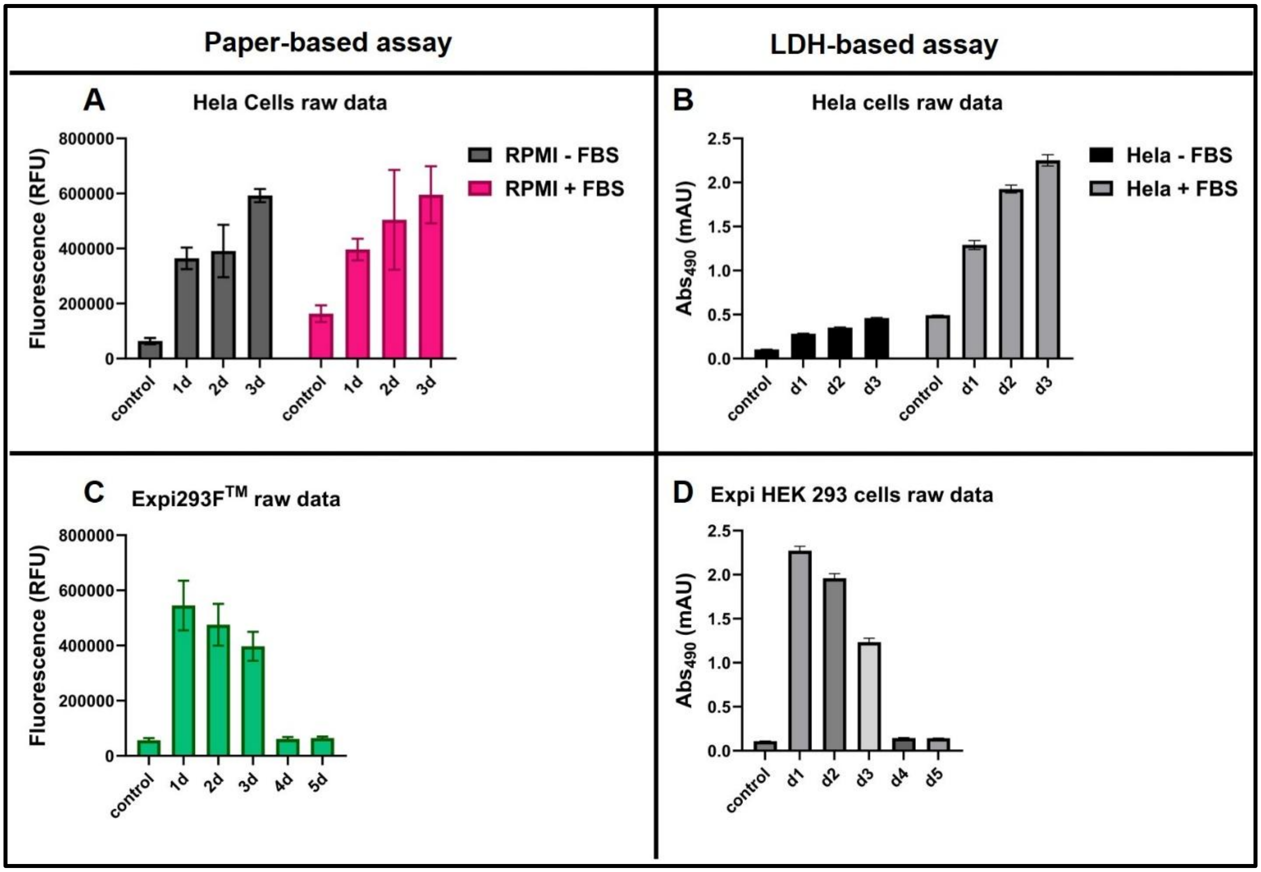

*Figure S 3: The raw fluorescence and absorbance data for the paper-based (**A, C**) and LDH-based (**B, D**) assay respectively, are shown. Error bars are the result of three individual measurements. Controls refer to sensors treated only with the respective medium. Cell culture supernatants were diluted 100-fold in the paper-based assay and 10-fold in the solution-based assay. MOPS buffer with 10 mM Ca^{2+} was used to dilute the samples.*

4 Analysis of the fiber-bound sensor after prolonged storage

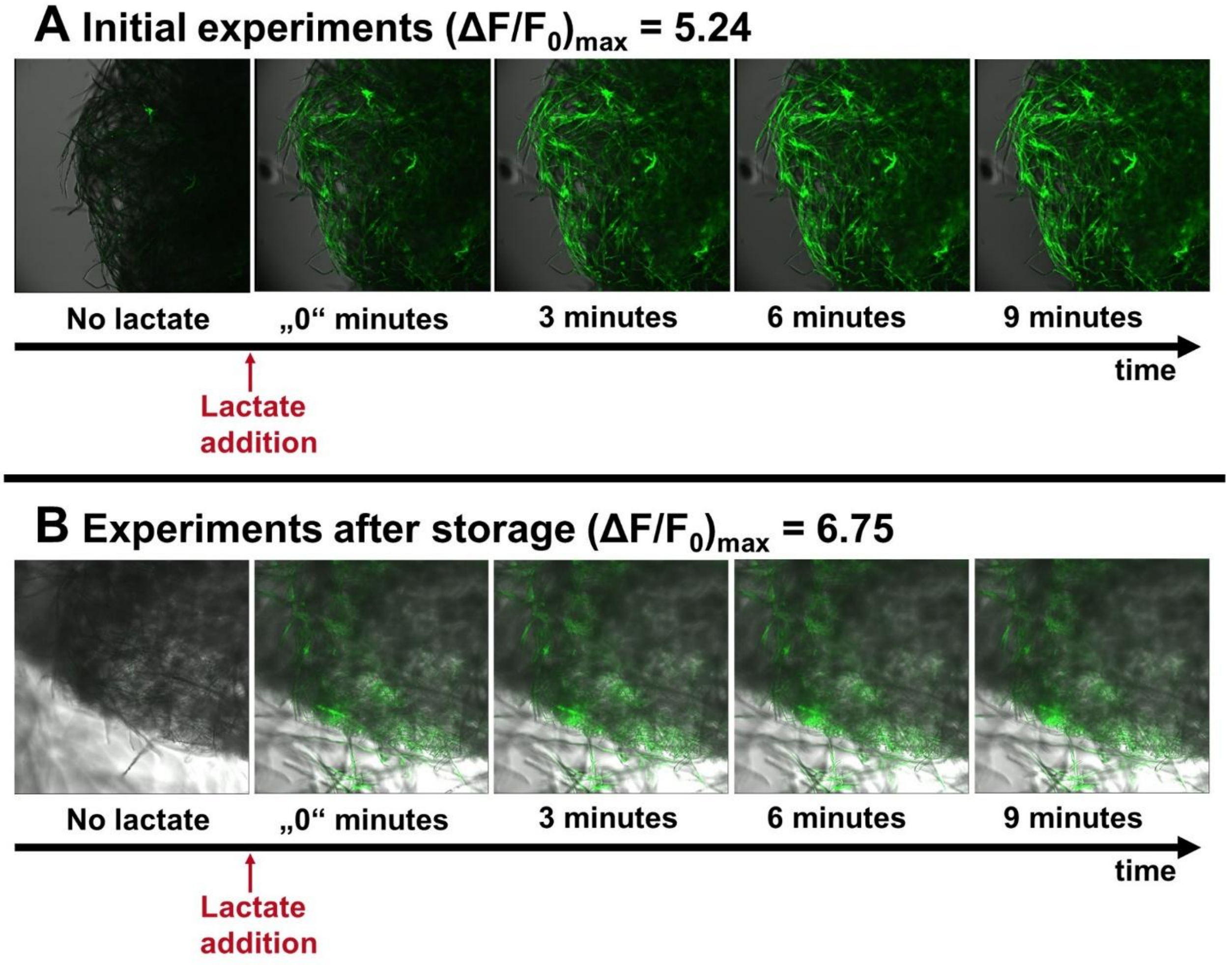

Figure S 4: Confocal microscopy images taken of sensor-modified eucalyptus fiber at different timepoints after lactate addition and the maximum $\Delta F/F_0$ – values determined by analyzing the mean fluorescence of the images in Fiji image J. ***A****: Initial data obtained within a few days of the modification of the fibers.* ***B****: Data obtained after prolonged storage of the fibers for 6 month.*

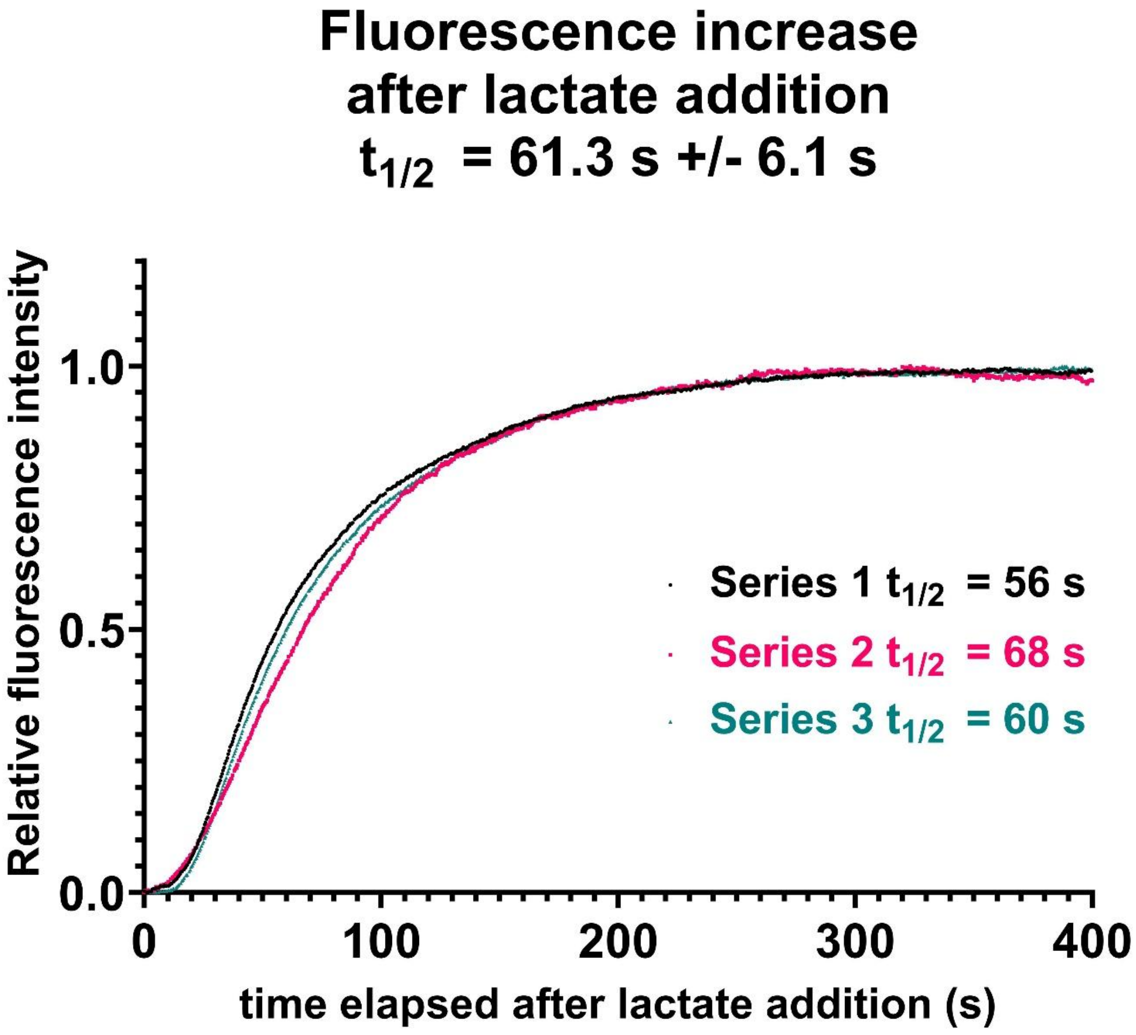

Figure S 5: The increase in fluorescence after lactate addition during the first 400 seconds is shown. 3 series were measured on different paper fibers to calculate the time at which the half-maximal fluorescence intensity is reached.
